# Long-Term Oncological Outcomes of Minimally Invasive Surgery in Non-Small Cell Lung Cancer: An Updated Review

**DOI:** 10.3390/cancers18050798

**Published:** 2026-02-28

**Authors:** Marco Donatello Delcuratolo, Michele Piazzolla, Doroty Sampietro, Lucia Anna Muscarella, Concetta Martina Di Micco, Antonella Centonza, Federico Pio Fabrizio, Domenico Trombetta, Franco Morelli, Francesco Passiglia, Paola Parente

**Affiliations:** 1 Medical Oncology Unit, Fondazione IRCCS Casa Sollievo della Sofferenza, 71013 San Giovanni Rotondo, FG, Italy; 2 Unit of Thoracic Surgery, Fondazione IRCCS Casa Sollievo della Sofferenza, 71013 San Giovanni Rotondo, FG, Italy; m.piazzolla@operapadrepio.it (M.P.);; 3 Laboratory of Oncology, Fondazione IRCCS Casa Sollievo della Sofferenza, 71013 San Giovanni Rotondo, FG, Italyd.trombetta@operapadrepio.it (D.T.); 4 Department of Medicine and Surgery, University of Enna “Kore”, 94100 Enna, EN, Italy; federicopio.fabrizio@unikore.it; 5 Department of Oncology, University of Turin, AOU S. Luigi Gonzaga, 10043 Orbassano, TO, Italy; 6 Unit of Pathology, Azienda Ospedaliera Universitaria Ospedali Riuniti, 71100 Foggia, FG, Italy; paolaparente77@gmail.com

**Keywords:** VATS, RATS, oncological outcomes, non-small cell lung cancer

## Abstract

Non-small cell lung cancer (NSCLC) accounts for approximately 85% of lung cancers, and surgical resection is the gold-standard treatment for resectable disease. Minimally invasive surgery (MIS), which includes video-assisted thoracoscopic surgery (VATS) and robotic-assisted thoracoscopic surgery (RATS), has emerged as an alternative option to thoracotomy, with the aim of minimizing perioperative morbidity without compromising oncological efficacy. This narrative review evaluates long-term oncological outcomes (overall survival (OS), disease-free survival (DFS) and recurrence-free survival (RFS)) in patients with NSCLC at different stages who underwent MIS. Retrospective and prospective studies, as well as meta-analyses, are included. VATS has shown comparable and, in many cases, superior oncological outcomes compared to open surgery, with more evident benefits in the early stages of the disease. Although mainly in retrospective studies, RATS has demonstrated efficacy in terms of oncological outcomes comparable to open surgery, even in advanced stages or complex resections. With regard to the direct comparison between VATS and RATS, the two MIS techniques have shown similar OS rates, albeit some prospective data and meta-analyses suggest a potential DFS advantage for RATS. MIS is a safe and effective surgical approach in terms of oncological outcomes for resectable NSCLC; nevertheless, it will be necessary to await the results of further randomized studies currently ongoing to better define the long-term benefits of each technique.

## Simple Summary

Surgery is the cornerstone of curative treatment for early-stage lung cancer. In recent years, minimally invasive surgery (VATS and RATS) has gradually replaced open thoracotomy, reducing morbidity, hospital stays, decreasing pain and speeding up postoperative recovery while maintaining oncological efficacy. This review provides an overview of the current evidence on long-term oncological outcomes after minimally invasive surgery, including disease-free/recurrence-free survival and overall survival. Although evidence based mainly on retrospective studies shows that minimally invasive approaches achieve oncological results comparable to open surgery, ongoing randomized studies will help to understand the best therapeutic strategy for patients with resectable NSCLC.

## 1. Introduction

Lung cancer is one of the most prevalent malignancies worldwide, being the leading cause of cancer mortality in men and the second one in women. Lung cancer incidence and mortality rate are affected by several factors: tobacco exposure is by far the main risk factor, whereas environmental exposures (such as air pollution, radon, and arsenic) have different weights depending on the geographical area. Moreover, genetic predisposition and immunodeficiency disorders (such as HIV infection) can contribute to lung cancer development [1].

Histologically, lung cancer is classified into two main types, small cell lung cancer (SCLC) and non-small cell lung cancer (NSCLC), with the latter accounting for 85% of the cases. NSCLC is further classified by histological subtype, the most common being adenocarcinoma (ADC) (40%), followed by squamous cell carcinoma (SCC) (25%) [2].

NSCLC is an aggressive disease with most cases detected at advanced stages, with only about 10% identified in stage I, affecting management strategy and prognosis. Moreover, survival rates dramatically decrease from stage I (75–90%) to stage IV (9%) [3]. In this setting, therapeutic approaches are driven by tumor clinical stage, tumor molecular profiling, and patient’s features.

In particular, in locally advanced/metastatic disease, medical systemic treatment is the recommended choice. In this setting, tumor molecular profiling and programmed death-ligand 1 (PD-L1) assessment are required in order to investigate the possibility of scheduling targeted therapy and immune checkpoint inhibitors (ICIs) with respect to conventional chemotherapy [4].

On the other hand, the surgical approach remains the backbone for early-stage resectable NSCLC patients, with a 5-year OS ranging from 60% to over 90% depending on the stage [5]. Moreover, the implementation of lung-cancer screening programs will certainly contribute to further increasing the earlier diagnosis of NSCLC, increasing the rate of patients suitable for surgical management [6].

The safety of the surgical approach, however, is affected by the patient’s age and comorbidities, such as cardiovascular disease and pulmonary function reduction, leading to great challenges among this frail population that are usually associated with a higher risk of postoperative complications [7,8].

In resectable NSCLC, open lobectomy (OL) with hilar and mediastinal lymph node dissection is associated with postoperative complications, deep pain and even mortalities, especially in elderly patients [9].

Less invasive surgery, such as atypical resection and sublobar resection, has shown similar outcomes in selected cases [9]. Moreover, these approaches already have a clinical indication in peripheral and small parenchymal nodules arising due to screening programs.

Recently, minimally invasive surgeries (MISs), combining technological supports like high-definition cameras and micro-instruments, have been developed leading to the performance of thoracic surgery via small incisions in the thoracic wall. These techniques have led to a reduction in postoperative complications and, thus, to an increase in patients suitable for a surgical approach. Moreover, MISs are also characterized by a shortened postoperative hospitalization, better perioperative outcomes and similar OS compared to thoracotomy in older patients [8]. The current standardized MISs are video-assisted thoracoscopic surgery (VATS) and robotic-assisted thoracoscopic surgery (RATS).

To maximize surgical treatment of NSCLC in every patient, such as the new concept of ‘surgical target-therapy’, and to achieve good long-term outcomes, it is imperative to fully understand the differences between the two techniques in order to be able to choose the optimal one according to the patient’s clinical features.

In this narrative literature review, we aim to describe the oncological outcomes of VATS and RATS, comparing the advantages and disadvantages as reported in the largest studies from the literature to provide updated insight into optimal surgical treatments in resectable NSCLC patients.

## 2. Methods

This narrative review aims to provide a broad overview of the long-term oncological outcomes of MIS in resectable NSCLC, including VATS and RATS, with a particular focus on OS and DFS/RFS. A literature search was performed through a non-systematic search on PubMed and the reference lists of key articles, prioritizing studies based on clinical relevance, methodological rigor, sample size, and representative coverage of the main surgical scenarios. Only articles related to NSCLC were considered. Only studies in English were considered, selecting from retrospective, prospective and meta-analysis studies. Studies that did not specifically focus on long-term oncological outcomes were not included, for example those whose objective was only related to perioperative outcomes.

## 3. Surgical Techniques: VATS and RATS for NSCLC

VATS lobectomy consists of small incisions (typically 3–5 ports), allowing for the insertion of a thoracoscope and long instruments into the thoracic cavity. The pulmonary artery, vein and bronchus of the lobe are individually dissected and stapled under video guidance, with specimens removed in an impermeable bag to prevent tumor seeding [10]. RATS employs a robotic platform (e.g., the da Vinci system) that offers high-definition 3D visualization and articulated instruments with greater degrees of motion and tremor filtering [11]. Typically 3–4 robotic ports are used, though emerging uniportal robotic-assisted thoracic surgery (U-RATS) allows for a single incision using advanced instruments, potentially improving cosmesis and reducing operative trauma [12]. The RATS system allows for precise dissection of the hilum and mediastinum, as well as systematic lymphadenectomy, which is particularly useful in the case of segmentectomy or for centrally located tumors [11,13].

Open thoracotomy remains the gold standard for complex resections requiring rib spreading or direct palpation. However, it causes greater chest wall trauma, postoperative pain and lengthens hospital stay (typically 7–10 days) [11,14,15].

Compared to open surgery, both VATS and RATS achieve equivalent oncologic radicality (microscopically margin-negative resection and R0 resection) with reduced morbidity, shorter hospital stay, decreased pain and quicker postoperative recovery [16,17,18].

With regard to the comparison between VATS and RATS, in the French EPITHOR multicenter registry, encompassing 5687 minimally invasive lung resections (3692 VATS and 1995 RATS) between 2016 and 2020, short-term outcomes and safety were similar between the two different techniques [19].

Several studies have consistently reported that RATS allows for a more extensive lymph node dissection, which may lead to improved pathologic staging [20,21].

Regarding technical advantages and learning curve, the RATS platform offers ergonomic improvements, three-dimensional visualization and wristed instrument control. This technical advantage may underlie more precise dissections and improved lymphadenectomy, especially in segmentectomies of small tumors [22].

In addition to the general population, particular interest has been focused on special subgroups of patients to whom the potential advantages of RATS may be more evident. For example, a French tertiary analysis of neoadjuvant chemo-immunotherapy in NSCLC demonstrated that RATS was associated with a higher number of N1 lymph nodes and stations harvested, as well as similar short-term outcomes compared to VATS [23]. In obese patients, pooled data from Society of Thoracic Surgeons (STS), Epithor and McMaster databases revealed significantly lower conversion rates to thoracotomy with RATS, along with shorter hospital stays and fewer respiratory complications when compared with VATS [24]. Pan et al. compared RATS, VATS and open surgery in patients aged 75 years or older. The results showed that RATS had the best surgical outcomes, including shorter operating time (*p* < 0.001) and less blood loss (*p* < 0.001). Furthermore, postoperative recovery was faster with RATS, including shorter intensive care unit stay (*p* = 0.004), postoperative hospital stay (*p* < 0.001), lower overall costs (*p* < 0.001) and lower incidence of postoperative complications (*p* = 0.002) [8].

A comparative overview of the main technical features of VATS, RATS and open thoracotomy is provided in Table 1.

## 4. Oncological Outcomes of Minimally Invasive Thoracic Surgery in NSCLC

There are numerous studies that have evaluated the oncological outcomes of MIS in NSCLC by comparing them with those of open surgery, without univocal conclusion [25,26,27,28,29,30,31,32].

This section primarily summarizes the long-term oncological results of VATS compared to open surgery, followed by evidence relating to RATS compared to open surgery and, finally, direct comparisons between VATS and RATS at different stages of the disease.

### 4.1. VATS

Several experiences have evaluated VATS in patients with different stages of NSCLC [33,34,35,36,37,38,39,40]. Gioutsos et al. demonstrated a 3-year OS of 87.9% in stage-IA patients undergoing uniportal-VATS (U-VATS) segmentectomy [33]. A study conducted by Marty-Ané et al. performed a retrospective analysis of 410 VATS lobectomies. It included 364 primary lung cancers, particularly early-stage NSCLC. The 3-year OS rates were 87.3% for stage IA, 76.5% for overall stage I, 58% for stage II and 61% for stage III; recurrence rates were 9.6% for stage I (mostly distant), 26.3% for stage II and 25% for stage III [36]. Another retrospective study by Wu et al. evaluated U-VATS in a sample of 307 patients with NSCLC at different stages. The 2-year DFS and 2-year OS, according to the American Joint Committee on Cancer (AJCC), 8th edition, were 92.3% and 100% for stage IA1, 73.7% and 91.4% for stage IA2, 75.2% and 93.4% for stage IA3, 62.1% and 85.9% for stage IB, 55.6% and 72.7% for stage IIA, 47.1% and 64.2% for stage IIB, and 42.1% and 60.3% for stage IIIA, respectively [38]. A prospective observational study by Luan et al. enrolled 109 patients with stages I, II or IIIA NSCLC who underwent VATS lobectomy. The OS rates after 1, 2, 3, 4 and 5 years were 100%, 85.9%, 65.3%, 55.9% and 55.9%, respectively. In multivariable analysis, only radical lymphadenectomy was an independent prognostic factor of survival (HR 3.94, 95% CI 1.41–11.0) [39]. Mun et al. included patients with an early stage (clinical, cT1-T2-T3 N0), showing different 5-year RFS between pathologic (p)N1 and pN2 who underwent multiportal-VATS (M-VATS) lobectomy [40].

No difference in long-term outcomes has been demonstrated between VATS-lobectomy and VATS-segmentectomy [41,42,43]. In particular, a study conducted by Lutz et al. using a ‘intent-to-treat’ prospective database, which included 621 stage-I patients, demonstrated, with a median follow-up of 34.5 months, a 5-year OS for the entire cohort of 75%, significantly different between clinical stages IA (76%) and IB (70.9%). Specifically, for neoplasms < 2 cm, no significant difference in OS was found between the segmentectomy and lobectomy groups (74% vs. 78.9%; *p* = 0.634) [43]. A meta-analysis by Bertolaccini et al. demonstrated the same results with no difference between MIS-segmentectomy and MIS-lobectomy in OS (*p* = 0.36) and DFS (*p* = 0.72) [44]. The same trend of comparable oncological outcomes was also found in a meta-analysis by Zeng [45], although Liu’s meta-analysis showed for stage I NSCLC an OS advantage of VATS-lobectomy over VATS-sublobectomy (*p* = 0.007) [46].

A meta-analyses and a PSM analysis showed no differences in oncological outcomes between U-VATS and M-VATS [47,48]. In particular, a meta-analysis by Sudarma et al. analyzed 13 PSM cohort studies comprising 3180 patients (U-VATS: 1587; M-VATS: 1593). With regard to long-term survival outcomes, it was shown that the probability of OS at 96 months was slightly higher in the M-VATS group (82.49%) than in the U-VATS group (75.89%), although not statistically significant (*p* = 0.5); while DFS was marginally higher in the U-VATS group (75.43%) than in the M-VATS group (74.74%), also not significant (*p* = 0.59) [47]. A second study by Zhou et al., using data from the Western China Lung Cancer Database (2014–2021), identified 2630 patients undergoing segmentectomy, and PSM was applied to balance baseline characteristics (uniportal: 400; three-port: 1200). With a median follow-up of 27 months, the oncological outcomes were similar between the two surgical techniques. Specifically, the 1-year OS was 100% for uniportal versus 99.9% for three-port (*p* = 0.36), the 3-year OS was 100% versus 90.4%, respectively (*p* = 0.20), while the 5-year OS was 97.7 and 99.4%, respectively (*p* = 0.78); progression-free survival (PFS) was also the same between the two groups [48].

Numerous studies have reported superior OS and RFS/DFS with VATS compared to open surgery, although others failed to show statistically significant differences. In a meta-analysis of 20 studies that included patients with stage I NSCLC, the 5-year OS rate of the VATS group was significantly higher than that of the thoracotomy group (OR 1.82, 95% CI 1.43–2.31; *p* < 0.01) [49]. Another meta-analysis included patients with NSCLC and pN2 disease; VATS improved OS at 3 years (HR 1.26, 95% CI 1.12–1.42; *p* = 0.0002) but with comparable 1-year DFS (HR 1.14, 95% CI 0.89–1.46; *p* = 0.32) and 3-year DFS (HR 1.03, 95% CI 0.88–1.22; *p* = 0.70) rates [50]. The same trend was reported in a meta-analysis by Taioli et al., which included 20 studies involving patients with stages I–II NSCLC [51], while Geropoulos et al., in a meta-analysis of six studies involving patients at various stages, found no differences in OS (*p* = 0.28) and RFS (*p* = 0.45) [52].

Other studies have evaluated VATS versus thoracotomy for early stages with contrasting results [53,54,55,56,57,58,59,60,61,62,63,64]. A retrospective cohort study evaluated the oncological outcomes of VATS lobectomy versus OL in patients > 65 years old with stage I lung cancer using Medicare-linked Society of Thoracic Surgeons data (2002–2013). This analysis, which involved 10,597 patients (VATS: 6149; thoracotomy: 4448), showed that those undergoing VATS generally had better health status at baseline, including lung function. A propensity-matched analysis showed a higher 4-year OS for VATS (68.6%) than for thoracotomy (64.8%), with statistical significance (*p* = 0.003) [54]. A propensity-matched study by Murakawa et al. explored the long-term efficacy of VATS lobectomy compared with OL in patients with cT1-2N0M0 NSCLC. The analysis showed that the VATS group had better survival for all oncologic outcomes in a statistically significant manner (*p* = 0.0049 for DFS, *p* = 0.0154 for disease-specific survival and *p* = 0.032 for OS). However, after comparing patients according to preoperative variables, the statistically significant difference was not confirmed, although the trend for better efficacy outcomes for VATS persisted [55]. In contrast, a retrospective study including 608 patients with stages I–II NSCLC who underwent lobectomy with VATS or open thoracotomy had no statistically significant differences between the two surgical techniques. Specifically, 5-year DFS was 69.1% versus 69.7% (*p* = 0.94), 5-year cancer-specific survival (CSS) was 82.9% versus 76.7% (*p* = 0.17), and 5-year OS was 73% versus 64% (*p* = 0.17) for the open and VATS groups, respectively [56]. Smith et al. demonstrated similar overall and lung CSS in elderly patients with stage I NSCLC treated with VATS or open segmentectomy [60]. Yun et al. end Nakano et al. showed no difference between VATS and thoracotomy in patients with T > 5 cm N0 [61,62], while another study showed an advantage in DFS but not in OS for VATS in cN0 adenocarcinoma patients [63]. Xie et al. found no differences between U-VATS and open-sleeve lobectomy for centrally located NSCLC patients [64]. Gao et al. detected no difference in OS between U-VATS and open pneumonectomy [65], although in another study by Al Sawalhi et al. OS appeared to be superior in U-VATS when pathology stage was aligned (*p* = 0.001) [66].

Numerous studies have evaluated the effectiveness of MIS in patients undergoing neoadjuvant treatment [67,68,69,70,71,72,73,74,75,76]. In a retrospective multicenter study by Dell’Amore et al., 286 patients with stages II, IIIA or IIIB undergoing VATS versus OL after neoadjuvant chemotherapy were evaluated; no difference in OS (*p* = 0.6) and DFS (*p* = 0.9) was reported [67]. This trend in terms of oncologic outcomes was confirmed by another study that included stage-IIIA N2 patients showing no differences in OS (*p* = 0.276) and RFS (*p* = 0.354) between the two surgical options [72]. Hireche’s meta-analysis also showed no difference in OS (*p* = 0.7) and DFS (*p* = 0.07) in this patient setting [73]. However, the study by Fang et al. demonstrated the superiority of VATS patients in terms of 3-year OS compared with thoracotomy (61% vs. 43%; *p* = 0.010) albeit only in univariate analysis, in fact not being confirmed after a PSM analysis (*p* = 0.56) [68].

Some studies have shown no difference in oncological outcomes between MIS and thoracotomy in patients treated with chemotherapy plus immunotherapy induction [70,74,75,76]. Pan et al. evaluated long-term oncologic outcomes in 143 patients with stage-IIIA and -IIIB NSCLC undergoing neoadjuvant therapy with chemotherapy plus immunotherapy. At a median follow-up of 29.5 months, VATS and thoracotomy demonstrated comparable rates of 2-year RFS (77.20% vs. 73.73%; *p* = 0.640) and OS (87.22% vs. 88.00%; *p* = 0.738) [74].

Other studies and meta-analyses that have compared VATS with open surgery in NSCLC patients are shown in Table 2 [77,78,79,80,81,82,83,84,85,86,87,88,89,90,91,92,93,94].

In summary, VATS has demonstrated oncological results comparable to and, in many cases, superior to open surgery, as evidenced in some meta-analyses [49,50,51], with more evident benefits in the early stages of the disease. It also appears to be safe and effective after neoadjuvant treatment.

### 4.2. RATS

A number of studies have evaluated the effectiveness of RATS in terms of oncological outcomes, in some cases in comparison with open surgery [95,96,97,98,99,100,101]. Cerfolio et al. studied the RATS approach in 1339 patients with NSCLC in different stages (from I to IIIB). The 5-year specific survival was 83% for stage-IA patients, 77% for stage-IB patients, 68% for stage-IIA patients, 70% for stage-IIB patients, 62% for stage-IIIA patients and 31% for stage-IIIB patients [95]. Another study retrospectively evaluated 104 patients with centrally located NSCLC who underwent single-sleeve bronchial lobectomy using RATS. Overall, the 5-year DFS and 5-year OS rates were 67.9% and 73%, respectively. In terms of pathological stage, the 5-year DFS and OS rates were 82.9% and 82.2%, respectively, for stage-I patients, 57.8% and 69.7% for stage-II patients, and 54.5% and 63.7% for stage-III patients; furthermore, multivariate analysis showed that a more advanced pathological stage or an N2 stage were independent risk factors for worse DFS and OS [96]. Another single-center retrospective analysis conducted on 500 patients undergoing RATS lobectomy showed a survival rate of 84% for stage-IA patients, 73% for stage-IB patients, 68% for stage-IIA patients, 63% for stage-IIB patients, and 49% for stage-IIIA patients [98].

Some studies have compared the RATS technique to open thoracotomy, showing no statistically significant differences in terms of long-term oncological outcomes. Zirafa et al. compared RATS versus open surgery lobectomy in 131 patients with stages IIIA–IIIB NSCLC. After a median follow-up of 70 months, the 5-year OS was 34% in the robotic group and 31% in the thoracotomy group. Specifically, after adjustment based on the T parameter, no differences emerged in terms of OS (*p* = 0.888), local recurrence (local recurrence-free survival, LRFS) (*p* = 0.562) and distant metastases (metastases-free survival, MFS) (*p* = 0.846) [100]. Another study by Gu et al. evaluated the oncological outcomes of patients with central lung carcinoma who underwent bronchial sleeve resection with a robotic or thoracotomic system. Also, in this case, no difference was demonstrated between the two approaches in terms of RFS (*p* = 0.16) and OS (*p* = 0.59). In the multivariate analysis, tumor size and postoperative radiotherapy were found to be significant predictors of RFS, while only intensive care unit stay was identified as a significant predictor of OS [101].

The available evidence, albeit from retrospective studies, indicates that RATS produces favorable oncological outcomes, with survival rates comparable to those of open surgery, even in advanced stages or complex resections, supporting its role as a valid alternative in selected patients. The studies comparing RATS and open surgery are shown in Table 3.

### 4.3. RATS vs. VATS (vs. Open)

Several studies have compared the RATS approach with VATS [20,23,102,103,104,105,106,107,108,109,110,111,112,113,114,115,116,117,118,119] and, in some cases, with open surgery [8,120,121,122,123,124,125,126,127,128].

Three studies [102,103,104] including one in an elderly population [104], were conducted in patients with stage IA NSCLC, showing no differences in OS and RFS/DFS between RATS and VATS. Another retrospective study was conducted on stage IIB-IIIA-IIIB patients undergoing neoadjuvant immunochemotherapy, showing similar results between the two surgical approaches in terms of RFS at 1 year (*p* = 0.821) [23]. Other retrospective studies confirmed the absence of differences between the two approaches and are summarized in Table 4 [105,106,107,108,109,110,111]. The only study that demonstrated an advantage in OS (*p* = 0.029) but not in DFS (*p* = 0.426) was conducted by Huang et al. [111].

When considering prospective experiences, Niu et al. demonstrated the same efficacy in terms of oncological outcomes in 320 patients from stages I to IIIA NSCLC. In this single-center, open-label, randomized and parallel-arm study, a total of 320 patients were randomized to receive RATS (n = 157) or VATS (n = 163). After a median follow-up of 58.0 months, the 3-year OS rate was 94.6% (95% CI 91.0–98.3) in the RATS group and 91.5% (95% CI 87.2–96.0) in the VATS group (HR 0.65, 95% CI 0.33–1.28; *p* = 0.21). Thus, the non-inferiority of RATS was confirmed based on a predefined margin of −5%; the 3-year DFS was 88.7% (95% CI 83.6–94.1) in the RATS group and 85.4% (95% CI 80.0–91.2) in the VATS group (HR 0.87, 95% CI 0.50–1.52; *p* = 0.62) [112]. A monocentric prospective trial that randomized 1 (RATS): 2 (VATS) patients from stage I to IIIA confirmed similar OS and DFS between the two groups [113]. However, another single-center prospective study conducted by Fabbri et al. on 619 patients showed a statistically significant difference in DFS between the RATS and VATS groups (3-year DFS: 92.4% vs. 81.2%; 5-year DFS: 90.3% vs. 77.6%; *p* < 0.001) although no significant difference in OS was observed (3-year OS: 75.9% vs. 82.3%; 5-year OS: 70.5% vs. 68.5%; *p* = 0.637). Furthermore, in the multivariate analysis, the surgical procedure was significantly associated with DFS, with an HR of 0.46 (95% CI 0.27–0.78, *p* = 0.004) for RATS compared to VATS [114]. In addition, two meta-analyses, involving patients who underwent segmentectomy or lobectomy using either VATS or RATS, confirmed the superiority of RATS over VATS in terms of DFS but not OS [116,117]. The first included 26 studies comprising more than 45,000 NSCLC patients, demonstrating a higher 5-year DFS for RATS (95% CI 1.11–2.57; *p* = 0.01) but a comparable 5-year OS (95% CI 0.90–1.02; *p* = 0.22) [116]. The second included 25 studies with 50,404 patients; the RATS group reported longer DFS than the VATS group (HR 0.76, 95% CI 0.59–0.97; *p* = 0.03) and a nonsignificant trend toward longer OS (HR 0.77, 95% CI 0.57–1.05; *p* = 0.10) [117]. However, meta-analyses conducted by Ma et al. and Mirza et al. did not identify any differences in the two oncological outcomes [118,119].

Other studies evaluated the differences in oncological outcomes between VATS, RATS and open surgery. With regard to stage-I disease, Yang et al. demonstrated 5y-OS rates of 77.6%, 73.5% and 77.9% for patients undergoing RATS, VATS and open respectively, with no statistically significant difference. In terms of 5y-DFS, RATS was superior to VATS (*p* = 0.047) but not to the open approach (*p* = 0.34) [120]. Casiraghi et al. examined 180 stage-I patients (n = 72 RATS; n = 36 VATS; n = 72 open); the 5-year survival was 78.6% for open surgery, 77.3% for VATS and 87.4% for RATS (*p* = 0.74 VATS; *p* = 0.20 RATS), while the 5-year CSS was 85.5%, 80.0% and 95.7%, respectively (*p* = 0.41, VATS; *p* = 0.15 RATS) [121]. Another retrospective analysis that included 1021 patients at different stages of disease did not identify any differences in OS between RATS versus VATS (*p* = 0.720) and between RATS versus Open (*p* = 0.953) [122].

Three meta-analyses compared RATS, VATS and open surgery [126,127,128]. Aiolfi’s meta-analysis included 34 studies involving a total of 183,426 patients. In particular, the 5-year OS was assessed in 16 studies (70,965 patients); no differences were found between RATS and open surgery (HR 0.61, 95% CI 0.32–1.08), VATS and open surgery (HR 0.93, 95% CI 0.73–1.19) and RATS and VATS (HR 1.53, 95% CI 0.87–2.88) [126]. Leitao’s meta-analysis (RECOURSE study) evaluated a comparison between VATS, RATS and open surgery for various types of malignancies (endometrial, cervical, colorectal, lung and prostate cancer); in particular for NSCLC lobectomy, DFS was superior for RATS over VATS (HR 0.74; *p* = 0.009) and OS favored RATS over open surgery (HR 0.93; *p* = 0.04) [127]. In Ng’s meta-analysis, (145 studies), M-VATS was superior compared to open surgery in terms of 5-year OS rates (71.5% vs. 66.7%; *p* < 0.001) while demonstrating the same outcomes when compared to the robotic group [128]. All studies comparing the three surgical approaches are included in Table 4 [8,120,121,122,123,124,125,126,127,128].

In conclusion, most retrospective studies fail to report significant differences between RATS and VATS in terms of OS, although prospective studies and meta-analyses show a potential advantage of RATS in terms of DFS. Furthermore, both minimally invasive approaches appear to be oncologically equivalent to open surgery in studies comparing the three surgical approaches, although in Leitao’s meta-analysis, OS was better for RATS than for open surgery, and DFS was better for RATS than for VATS [127].

## 5. Conclusions and Future Perspectives

Minimally invasive surgery has become an established oncological standard for resectable NSCLC. VATS yields long-term oncological results comparable to thoracotomy and, in several series and meta-analyses, shows improved overall survival in early-stage disease while preserving the well-known perioperative advantages of reduced surgical trauma and shorter hospital stays. Robotic approaches replicate these long-term oncological outcomes and add technical refinements that not only ensure more accurate lymphadenectomy and improved pathological staging but also enhance safety, thus representing a great opportunity for special patient subgroups. RATS may provide particular advantages in obese patients, patients with complex hilar or mediastinal anatomy, patients undergoing anatomical segmentectomies or post-neoadjuvant chemotherapy-immunotherapy, and elderly or frail patients, in whom lower conversion rates and improved technical control have been reported. Direct comparisons between RATS and VATS generally show equivalent OS; however, prospective data and meta-analyses show an advantage in terms of DFS/RFS for RATS, suggesting a potential impact on micrometastasis control. Both MIS techniques have been shown to maintain oncological benefits after complex resections when performed by experienced surgical teams and also, especially, VATS after neoadjuvant chemoimmunotherapy treatment.

However, there are several limitations to the scientific evidence currently available (bias in patient selection, heterogeneity in staging and adjuvant strategies and scarcity of randomized studies), meaning that the superiority of MIS over open surgery in terms of oncological outcomes is not yet definitive and clear. Furthermore, current evidence suggests that RATS may be associated with improved disease-free survival in selected settings; however, these findings remain hypothetical and do not establish superiority over VATS. Randomized and prospective studies currently ongoing (Table 5) are designed to clarify the magnitude of survival differences among different techniques.

As highlighted in Karuppannan’s editorial on the randomized RVlob trial that currently has the most robust data [112], longer follow-up (5–10 years) is needed to determine whether a difference in oncological outcomes emerges over time [129]. However, in the real world, the choice between different minimally invasive surgical techniques should consider fundamental practical aspects, such as cost-effectiveness, institutional resources, and the learning curve associated with advanced technologies. In fact, RATS offers technical advantages but at the expense of higher costs and reduced current availability, effectively limiting its widespread adoption, especially in resource-poor settings. However, a strategic planning approach could include centralizing case volume and standardized perioperative pathways, which would allow for the initial investment to be gradually offset over time, while maintaining high safety standards.

Surgical experience remains the key factor in determining outcomes for both VATS and RATS, it being understood that all surgical decision making must be discussed in a multidisciplinary setting.

In recent years, the advent of neoadjuvant and perioperative chemoimmunotherapy has changed the therapeutic landscape of early-stage NSCLC. Several randomized trials have demonstrated significant improvements in pathological complete response (pCR), DFS and, in some studies, OS in patients with resectable stage-II and -III NSCLC [130,131,132,133]. In the adjuvant setting, both immunotherapy and target therapies (for EGFR- and ALK-positive patients) have also shown significant survival benefits in selected populations [134,135,136,137]. In this evolving scenario, the interpretation and evaluation of long-term oncological outcomes achieved with different surgical approaches should be contextualized within the broader framework of perioperative systemic therapies, as these treatments may independently influence survival endpoints.

To date, therefore, the choice of surgical approach should be personalized, taking into account the requirement for radical resection, the patient’s comorbidities, the surgeon’s experience and perioperative systemic therapies.

Overall, the data confirm that MIS is a safe and effective paradigm with the potential to improve oncological outcomes in resectable NSCLC.

## Figures and Tables

**Table 1:** VATS vs. RATS vs. open thoracotomy: surgical approach overview.

Feature	VATS	RATS	Open Thoracotomy
Incisions	3–5 ports	3–4 ergonomic robotic ports (or uniportal option)	Single large incision (10–20 cm) with rib spreading
Visualization	2D, 30° scope	3D high-definition, depth perception	Direct vision (natural 3D), headlight assistance
Instrument dexterity	Straight instruments, limited articulation	Wristed, tremor-filtered, enhanced maneuverability	Full manual articulation, tactile feedback
Lymphadenectomy	Competent but reliant on surgeon skill	Often more thorough, especially in mediastinal zones	Historical gold standard, extensive access to all nodal stations
Learning curve	Steep for lobectomy, especially uniportal	Faster for complex dissections after initial volume	Traditional training standard; technically demanding but widely established
Hospital stay & post-op pain	Shorter than open thoracotomy	Similar or slightly shorter than VATS in experienced centers	Longer hospital stay, higher post- operative pain
Contraindications	Large central tumors, dense adhesions	Similar but may handle more complex anatomy efficiently	Few absolute contraindications; preferred for very large tumors, chest wall invasion, or complex resections
Limitations	2D visualization, limited instrument articulation, reduced tactile feedback, technically demanding in complex resections or post-induction fibrosis	Higher costs, longer setup time, lack of haptic feedback, limited availability and dependence on institutional volume/experience	Greater surgical trauma, higher postoperative pain, longer hospital stay, slower functional recovery

**Table 2:** Oncological outcomes of VATS in NSCLC patients.

Authors (References)	Population	Surgery	Outcomes
Gioutsos et al. [33]	Stage IA; 85 pts	U-VATS Segmentectomy	3y-OS 87.9%
Jalil et al. [34]	Stage not specified; 69 pts	U-VATS	3y-OS 91.3%; 3y-DFS 87%
Kang et al. [35]	Stages I–II–III–IV; 170 pts	U-VATS Segmentectomy, Lobectomy, Bilobectomy	DFS 66.3 m; OS 67 m (total population)
Marty-Ané [36]	Stages I–II–III; 410 pts	VATS Lobectomy	3y-OS 76.5% (I); 58% (II), 61% (III)
Okada et al. [37]	Stage I; 102 pts	VATS Segmentectomy	5y-OS 89.8%; 5y-DFS 84.7%
Wu et al. [38]	Stages I–II–IIIA; 307 pts	U-VATS various surgical procedures	2y-OS 100% (IA1), 60.3% (IIIA); 2y-DFS 92.3% (IA1), 42.1% (IIIA)
Luan et al. [39]	Stages I–II–IIIA; 109 pts	VATS Lobectomy	3y-OS 65.3% (total population)
Mun et al. [40]	cT1-T2-T3 N0; 660 pts	M-VATS Lobectomy	5y-OS 88.1% (pN1), 80% (pN2); 5y-RFS 63.9% (pN1), 34.8% (pN2)
Helminen et al. [41]	Stages I–II–IIIA–IIIB; 215 pts	VATS-Segmentectomy vs. VATS-Lobectomy	No difference in 3y-OS (*p* = 0.412) and 3y-RFS (*p* = 0.450)
Song et al. [42]	Stage IA; 163 pts	VATS-Segmentectomy vs. VATS-Lobectomy	No difference in DFS (*p* = 0.157)
Lutz et al. [43]	Stage I; 621 pts	VATS-Segmentectomy vs. VATS-Lobectomy	No difference in OS (*p* = 0.634)
Bertolaccini [44]	Meta-analysis (10 studies); stage IA	MIS-Segmentectomy vs. MIS-Lobectomy	No difference in OS (*p* = 0.36) and DFS (*p* = 0.72)
Zeng et al. [45]	Meta-analysis (12 studies); stage I	VATS-Segmentectomy vs. VATS-Lobectomy	No difference in OS (*p* = 0.36) and DFS (*p* = 0.39)
Liu et al. [46]	Meta-analysis (8 studies); stage I	VATS-Sublobectomy vs. VATS-Lobectomy	OS better for VATS-lobectomy (*p* = 0.007)
Sudarma et al. [47]	Meta-analysis (13 studies); stages I–II–III	U-VATS vs. M-VATS Segmentectomy, Lobectomy	No difference in OS (*p* = 0.5) and DFS (*p* = 0.59)
Zhou et al. [48]	Stage I; 2630 pts	U-VATS vs. M-VATS Segmentectomy	No difference in OS (*p* = 0.784) and PFS (*p* = 0.180)
Chen et al. [49]	Meta-analysis (20 studies); stage I	VATS vs. Open Lobectomy	5y-OS better for VATS (*p* < 0.01)
Li et al. [50]	Meta-analysis (10 studies); pN2	VATS vs. Open Lobectomy	3y-OS better for VATS (*p* = 0.0002); No difference in 3y-DFS (*p* = 0.70)
Taioli et al. [51]	Meta-analysis (20 studies); stages I–II	VATS vs. Open Lobectomy	5y-OS better for VATS (meta difference in survival: 5%, 95% CI 3–6%)
Geropoulos [52]	Meta-analysis (6 studies); stages I–II–III–IV	VATS vs. Open-sleeve Lobectomy	No difference in OS (*p* = 0.28) and RFS (*p* = 0.45)
Liu et al. [53]	Stage I; 212 pts	VATS vs. Open Lobectomy	No difference in OS (*p* = 0.624) and DFS (*p* = 0.988)
Boffa et al. [54]	Stage I; 10,597 pts with ≥65y	VATS vs. Open Lobectomy	4y-OS better for VATS (*p* = 0.003)
Murakawa et al. [55]	cT1-T2 N0; 285 pts	VATS vs. Open Lobectomy	OS (*p* = 0.032) and DFS (*p* = 0.0049) better for VATS
Hanna et al. [56]	Stages I–II; 608 pts	VATS vs. Open Lobectomy	No difference in OS (*p* = 0.17) and DFS (*p* = 0.94)
Stephens et al. [57]	Stage I; 963 pts	VATS vs. Open Lobectomy	No difference in OS (*p* = 0.071)
Ghaly et al. [58]	Stage I; 193 pts	VATS vs. Open Segmentectomy	5y-OS (*p* = 0.017) and 5y-DFS (*p* = 0.013) better for VATS
Higuchi et al. [59]	Stage IA; 160 pts	VATS vs. Open Lobectomy	5y-OS better for VATS (*p* = 0.02); No difference in 5y-DFS (*p* = 0.15)
Smith et al. [60]	Stage I; 577 pts with ≥65y	VATS vs. Open Segmentectomy	No difference in OS (HR 0.80, 95% CI 0.6–1.06)
Yun et al. [61]	cT > 5 cm N0; 355 pts	VATS vs. Open Lobectomy	No difference in OS (*p* = 0.390) and RFS (*p* = 0.21)
Nakano et al. [62]	cT > 5 cm N0; 68 pts	VATS vs. Open Lobectomy	No difference in OS (*p* = 0.48)
Yamashita et al. [63]	cN0 adenocarcinoma; 240 pts	VATS vs. Open Lobectomy, Segmentectomy	DFS better for VATS (*p* = 0.04); No difference in OS (*p* = 0.58)
Xie et al. [64]	Centrally located NSCLC; 133 pts	U-VATS vs. Open-sleeve Lobectomy	No difference in OS (*p* = 0.81) and RFS (*p* = 0.78)
Gao et al. [65]	Stages IB–II–IIIA–IIIB; 850 pts	U-VATS vs. Open Pneumonectomy	No difference in OS (*p* = 0.9)
Al Sawalhi et al. [66]	Stages I–II–IIIA–IIIB; 318 pts	U-VATS vs. Open Pneumonectomy	OS better for U-VATS when pathology stage was aligned (*p* = 0.001)
Dell’Amore et al. [67]	Stages II–IIIA–IIIB; 286 pts	VATS vs. Open Lobectomy after CT	No difference in OS (*p* = 0.6) and DFS (*p* = 0.9)
Fang et al. [68]	Stages I–II–III–IV; 81 pts; squamous histology	VATS vs. Open various surgical procedures after CT	No difference in OS (*p* = 0.925) and DFS (*p* = 0.335)
Yang et al. [69]	Stages IB–II–III–IV; 272 pts	VATS vs. Open Lobectomy after CT	OS better for VATS (*p* = 0.01); No difference RFS (*p* = 0.12)
Hireche et al. [70]	Stages IIIA–IIIB; 205 pts	VATS vs. Open Lobectomy after CT/CT + IO/CRT	No difference in OS (*p* = 0.622) and RFS (*p* = 0.355)
Jeon et al. [71]	Stages IIIA N2; 35 pts	VATS vs. Open various surgical procedures after CRT	No difference in OS (*p* = 0.39) and DFS (*p* = 0.8)
Jeon et al. [72]	Stages IIIA N2; 385 pts	VATS vs. Open Lobectomy after CRT	No difference in OS (*p* = 0.276) and RFS (*p* = 0.354)
Hireche et al. [73]	Meta-analysis (9 studies); stages IIB–III–IV	VATS vs. Open Lobectomy after CT/CRT	No difference in OS (*p* = 0.7) and DFS (*p* = 0.07)
Pan et al. [74]	Stages IIIA–IIIB; 143 pts	VATS vs. Open Lobectomy after CT + IO	No difference in OS (*p* = 0.738) and RFS (*p* = 0.640)
Zhang et al. [75]	Stages IB–II–IIIA–IIIB; 131 pts	VATS vs. Open various surgical procedures after CT + IO	No difference in RFS (*p* = 0.204)
Deng et al. [76]	Stage IIIB; 31 pts	MIS (various surgical procedures) after CT + IO	DFS 27.5 m
Berry et al. [77]	Stages I–II–III–IV; 1087 pts	VATS vs. Open Lobectomy	5y-OS better for VATS (*p* < 0.001)
Li et al. [78]	Stages I–II–III; 6405 pts	VATS vs. Open various surgical procedures	5y-OS (*p* < 0.001) and 5y-RFS (*p* = 0.003) better for VATS
Tanase et al. [79]	Stages I–II–IIIA–IIIB; 84 pts	VATS vs. Open Lobectomy	No difference in OS (*p* = 0.447)
Witte et al. [80]	Stages I–II–III; 100 pts	VATS vs. Open Segmentectomy	5y-OS better for VATS (*p* = 0.047) No difference in 5y-RFS (*p* = 0.48)
Zhong et al. [81]	cN0-pN2; 157 pts	VATS vs. Open Lobectomy	No difference in OS (*p* = 0.45) and DFS (*p* = 0.46)
Chen et al. [82]	Stages II–IIIA; 240 pts	VATS vs. Open Lobectomy	No difference in OS (*p* = 0.73) and DFS (*p* = 0.40)
Paul et al. [83]	Stages I–II–III–IV; 6008 pts	VATS vs. Open Lobectomy	No difference in OS (*p* = 0.55) and DFS (*p* = 0.46)
Lee et al. [84]	Stages I–II–III; 416 pts	VATS vs. Open Lobectomy	No difference in OS (*p* = 0.767) and DFS (*p* = 0.890)
Merritt et al. [85]	cN0; 129 pts	VATS vs. Open Lobectomy	No difference in 3y-OS (*p* = 0.60)
Liu et al. [86]	pN2; 1034 pts	VATS vs. Open Lobectomy	No difference in OS (*p* = 0.821) and DFS (*p* = 0.890)
Yun et al. [87]	cN1; 1149 pts	VATS vs. Open Lobectomy	No difference in OS (*p* = 0.588) and RFS (*p* = 0.651)
Nakao et al. [88]	cT1-2-3-4 cN0-1-2; 1166 pts	VATS vs. Open Lobectomy	5y-OS better for VATS (*p* < 0.0001)
Nitsche et al. [89]	Stages I–II–III; 108 pts	VATS vs. Open- sleeve Lobectomy, Pneumonectomy	No difference in OS (*p* = 0.053)
Lim et al. [90]	cT1-2-3-cN0-1; 503 pts (randomized 1:1)	VATS vs. Open Lobectomy	No difference in OS (HR 0.67) and PFS (HR 0.74)
Dittberner et al. [91]	Stages I–II–IIIA–IIIB; 192 pts (randomized 1:1)	VATS vs. Open Lobectomy	No difference in OS (*p* = 0.29) and DFS (*p* = 0.17)
IJsseldijk et al. [92]	Meta-analysis (17 studies); stage IIIA	VATS vs. Open Lobectomy	No difference in OS and DFS
Deng et al. [93]	Meta-analysis (5 studies); centrally located NSCLC	VATS vs. Open sleeve Lobectomy	No difference in OS (*p* = 0.23) and PFS (*p* = 0.13)
Cai et al. [94]	Meta-analysis (6 studies); centrally located NSCLC	VATS vs. Open sleeve Lobectomy	No difference in OS (*p* = 0.58) and DFS (*p* = 0.35)

Abbreviations: NSCLC: non-small cell lung cancer; pts: patients; OS: overall survival; RFS: recurrence-free survival; DFS: disease-free survival; y: years; m: months; VATS: video-assisted thoracic surgery; MIS: minimally invasive surgery; U-VATS: uniportal VATS; M-VATS: multiportal VATS; CT: chemotherapy; IO: immunotherapy; CRT: chemoradiotherapy; c: clinical; *p*: pathological; HR: hazard ratio.

**Table 3:** Oncological outcomes of RATS in NSCLC patients.

Authors (References)	Population	Surgery	Outcomes
Cerfolio et al. [95]	Stages I–II–IIIA–IIIB; 1339 pts	RATS Lobectomy	5y-OS: 83% (IA), 77% (IB), 68% (IIA), 70% (IIB), 62% (IIIA), 31% (IIIB)
Liu et al. [96]	Stages I–II–III; 104 pts	RATS sleeve Lobectomy	5y-OS: 82.2% (I), 69.7% (II), 63.7% (III)
Zirafa et al. [97]	Stages I–II–III–IV; 212 pts	RATS Lobectomy	OS: 82 m (I), 73.5 m (II), 61.4 m (III), 41.3 m (IV)
Herrera et al. [98]	Stages I–II–III–IV; 500 pts	RATS Lobectomy	5y-OS: 84% (IA), 73% (IB), 68% (IIA), 63% (IIB), 49% (IIIA)
Park et al. [99]	Stages I–II–IIIA; 325 pts	RATS Lobectomy	5y-OS: 91% (IA), 88% (IB), 49% (II); 3y-OS: 43% (IIIA)
Zirafa et al. [100]	Stage IIIA–IIIB; 131 pts	RATS vs. Open Lobectomy	No difference in OS (*p* = 0.888), LRFS (*p* = 0.562) and MFS (*p* = 0.846)
Gu et al. [101]	Centrally located NSCLC; 103 pts	RATS vs. Open sleeve resection	No difference in OS (*p* = 0.59) and RFS (*p* = 0.16)

Abbreviations: NSCLC: non-small cell lung cancer; pts: patients; OS: overall survival; RFS: recurrence-free survival; LRFS: local recurrence-free survival; MFS: metastases-free survival; y: years; m: months; RATS: robotic-assisted thoracic surgery.

**Table 4:** Oncological outcomes of RATS vs. VATS (vs. Open) in NSCLC patients.

Authors (References)	Population	Surgery	Outcomes
Zhou et al. [102]	Stage IA; 130 pts	RATS vs. VATS Segmentectomy	No difference in OS (*p* = 0.642) and RFS (*p* = 0.144)
Forcada et al. [103]	Stage IA; 321 pts	RATS vs. VATS Segmentectomy, Lobectomy, Bilobectomy	No difference in OS (*p* = 0.848) and DFS (*p* = 0.117)
Pan et al. [104]	Stage IA; 594 octogenarians pts	RATS vs. VATS Wedge resection, Segmentectomy	No difference in OS (*p* = 0.891) and RFS (*p* = 0.782)
Pan et al. [23]	Stage IIB–IIIA–IIIB; 46 pts	RATS vs. VATS Lobectomy after CT + IO	No difference in 1y-RFS (*p* = 0.821)
Montagne et al. [105]	Stages I–II–III–IV; 844 pts	RATS vs. VATS Segmentectomy, Lobectomy	Lobectomy: no difference in 5y-DFS (*p* = 0.24) and 5y-OS (*p* = 0.084); Segmentectomy: no difference in 3y-DFS (*p* = 0.21) and 3y-OS (*p* = 0.51)
Shahoud et al. [106]	Stage not specified; 128 pts	U-VATS vs. RATS	No difference in OS and RFS (*p* > 0.05)
Merritt et al. [107]	cT1-T2-T3 N0-N1-N2 and stage IV; 200 pts	RATS vs. VATS Lobectomy	No difference in OS (*p* = 0.097) and RFS (*p* = 0.769)
Agyabeng-Dadzie et al. [108]	Stages I–II–IV; 478 pts	RATS vs. VATS Segmentectomy	No difference in 3y-OS (*p* = 0.11) and 3y-DFS (*p* = 0.40)
Haruki et al. [109]	Stages I–II or more; 299 pts	RATS vs. VATS Lobectomy	No difference in 3y-RFS (*p* = 0.21)
Sesti et al. [110]	Stages I–II–III–IV; 4307 pts	RATS vs. VATS Lobectomy	No difference in 3y-OS (*p* = 0.924)
Huang et al. [111]	Stage not specified; 166 pts	RATS vs. VATS Lobectomy	No difference in DFS (*p* = 0.426); OS better for RATS (*p* = 0.029)
Niu et al. [112]	Stages I–II–IIIA; 320 pts (randomized trial)	RATS vs. VATS Lobectomy	No difference in OS (*p* = 0.21) and DFS (*p* = 0.62)
Catelli et al. [113]	Stages I–II–IIIA; 75 pts (randomized trial)	RATS vs. VATS Lobectomy	No difference in OS (*p* = 0.461) and DFS (*p* = 0.312)
Fabbri et al. [114]	Stages I–II–III; 619 pts	RATS vs. VATS Lobectomy	No difference in OS (*p* = 0.637); DFS better for RATS (*p* < 0.001)
Nakamura et al. [115]	Stages II–IIIA–IIIB; 145 pts	RATS vs. VATS Lobectomy	No difference in OS (*p* = 0.30) and DFS (*p* = 0.12)
Zhang et al. [116]	Meta-analysis (26 studies); stage not specified	RATS vs. VATS Segmentectomy, Lobectomy	No difference in OS (*p* = 0.22); DFS better for RATS (*p* = 0.01)
Wu et al. [117]	Meta-analysis (25 studies); stage not specified	RATS vs. VATS Segmentectomy, Lobectomy	No difference in OS (*p* = 0.10); DFS better for RATS (*p* = 0.03)
Ma et al. [118]	Meta-analysis (18 studies); Stages I–II–III–IV	RATS vs. VATS Segmentectomy, Lobectomy	No difference in OS (*p* = 0.880) and DFS (*p* = 0.890)
Mirza et al. [119]	Meta-analysis (4 randomized studies); stage not specified	RATS vs. VATS Lobectomy	No difference in OS (HR 0.64, 95% CI 0.34–1.23) and DFS (HR 0.83, 95% CI 0.48–1.42)
Zhang et al. [20]	Stage I; 518 pts	RATS vs. VATS Lobectomy	No difference in OS (*p* = 0.62) and RFS (*p* = 0.70)
Yang et al. [120]	Stage I; 470 pts	RATS vs. VATS vs. Open Lobectomy	5y-OS: 77.6% (RATS) and 73.5% (VATS) (*p* = 0.10), 77.9% (Open) (*p* = 0.53 Open vs. RATS); 5y-DFS: 72.7% (RATS) and 65.5% (VATS) (*p* = 0.047), 69% (Open) (*p* = 0.34 Open vs. RATS)
Casiraghi et al. [121]	Stage I; 180 pts	RATS vs. VATS vs. Open Lobectomy	5y-OS: 87.4% (RATS) and 77.3% (VATS) 78.6% (Open) (*p* = 0.74 VATS and *p* = 0.20 RATS)
Baldonado et al. [122]	Stages I–II–III–IV; 1021 pts	RATS vs. VATS vs. Open Lobectomy	No difference in OS RATS vs. VATS (*p* = 0.720) and RATS vs. Open (*p* = 0.953)
Kneuertz et al. [123]	Stages I–II–IIIA; 514 pts	RATS vs. VATS vs. Open Lobectomy	No difference in 5y-OS (*p* = 0.56)
Qiu et al. [124]	Centrally located NSCLC; 188 pts	RATS vs. VATS vs. Open sleeve Lobectomy	No difference in OS and DFS (*p* > 0.05)
Shah et al. [125]	Stages I–II–III–IV; 3785 pts	RATS vs. VATS vs. Open Pneumonectomy	No difference in OS (*p* = 0.560)
Aiolfi et al. [126]	Meta-analysis (34 studies); Stages I–II–III–IV	RATS vs. VATS vs. Open Lobectomy	No difference in 5y-OS (*p* > 0.05)
Leitao et al. [127]	Meta-analysis (11 studies); stage not specified	RATS vs. VATS vs. Open Lobectomy	OS better for RATS over Open (*p* = 0.04); DFS better for RATS over VATS (*p* = 0.009)
Ng et al. [128]	Meta-analysis (145 studies); stage not specified	RATS vs. VATS vs. Open Lobectomy	5y-OS better for VATS vs. Open (*p* < 0.001)
Pan et al. [8]	Stages I–II–IIIA–IIIB; 504 pts aged 75y or older	RATS vs. VATS vs. Open Lobectomy	No difference in OS (*p* = 0.704) and DFS (*p* = 0.574)

Abbreviations: NSCLC: non-small cell lung cancer; OS: overall survival; RFS: recurrence-free survival; DFS: disease-free survival; y: years; VATS: video-assisted thoracic surgery; U-VATS: uniportal VATS; RATS: robotic-assisted thoracic surgery; CT: chemotherapy; IO: immunotherapy; c: clinical; HR: hazard ratio.

**Table 5:** Interventional randomized ongoing trials (with known status) evaluating long-term oncological outcomes of MIS in NSCLC.

NCT Number (Name)	Treatment Arms	Oncological Endpoints	N (Estimated Enrollment)	Status	Estimated Study Completion Date
NCT06202690 (SPORTS)	VATS vs. RATS Segmentectomy or Lobectomy, stages cI–II–IIIA	5y-DFS, 5y-OS	290	Not yet recruiting	30 June 2031
NCT03786003 (ECTOP-1007)	VATS vs. Open Lobectomy, stage cT1N0M0	3y-DFS (primary endpoint), 3y-OS	1086	Recruiting	2 September 2028
NCT06646770 (SELTIC)	Segmentectomy vs. Lobectomy (VATS, RATS and Open), stage cT1c	5y-RFS (primary endpoint), 5y-OS (primary endpoint)	400	Not yet recruiting	January 2030
NCT02617186	VATS vs. RATS Lobectomy, stages cI–II–IIIA	5y-OS	446	Active, not recruiting	September 2031
NCT06524427 (RAVAR)	VATS vs. RATS Lobectomy, stages cI–II	5y-DFS (primary endpoint), 3y-DFS, 5y-OS, 3y-OS	1124	Recruiting	31 December 2030

Clinicaltrials.gov. Abbreviations: OS: overall survival; RFS: recurrence-free survival; DFS: disease-free survival; y: years; VATS: video-assisted thoracic surgery; RATS: robotic-assisted thoracic surgery; c: clinical.

## Data Availability

No new data were created or analyzed in this study.

## Abbreviations

The following abbreviations are used in this manuscript: **ADC**: adenocarcinoma; **AJCC**: American Joint Committee on Cancer; **CI**: confidence interval; **CRT**: chemoradiotherapy; **CT**: chemotherapy; **CSS**: cancer-specific survival; **DFS**: disease-free survival; **HR**: hazard ratio; **ICIs**: immune checkpoint inhibitors; **IO**: immunotherapy; **LRFS**: local recurrence-free survival; **MFS:** metastases-free survival; **MIS**: minimally invasive surgery; **M-VATS**: multiportal video-assisted thoracoscopic surgery; **NSCLC**: non-small cell lung cancer; **OL**: open lobectomy; **OR:** odds ratio; **OS**: overall survival; **PD-L1**: programmed death-ligand 1; **PFS**: progression-free survival; **PSM**: propensity score matching; **pts**: patients; **R0**: microscopically margin-negative resection; **RATS**: robotic-assisted thoracoscopic surgery; **RFS**: recurrence-free survival; **SCC**: squamous cell carcinoma; **SCLC**: small cell lung cancer; **STS**: Society of Thoracic Surgeons; **U-RATS**: uniportal robotic-assisted thoracic surgery; **U-VATS**: uniportal video-assisted thoracoscopic surgery; **VATS**: video-assisted thoracoscopic surgery; **y**: years; **m**: months; **c**: clinical; ***p***: pathological.
